# Switching to Long-Acting Cabotegravir and Rilpivirine in Turkey: Perspectives from People Living with HIV in a Setting of Increasing HIV Incidence

**DOI:** 10.3390/medicina61081373

**Published:** 2025-07-29

**Authors:** Rıdvan Dumlu, Yeliz Çiçek, Mahir Kapmaz, Okan Derin, Halis Akalın, Uğur Önal, Egemen Özdemir, Çiğdem Ataman Hatipoğlu, Günay Tuncer Ertem, Alper Şener, Leyla Akgül, Yeşim Çağlar, Derya Tuna Ecer, Mustafa Kemal Çelen, Nur Bahar Oğuz, Figen Yıldırım, Deniz Borcak, Sevtap Şenoğlu, Eyüp Arslan, Sinan Çetin, Meryem Balcı, Ali Mert

**Affiliations:** 1 Department of Infectious Diseases and Clinical Microbiology, Faculty of Medicine, Istanbul Medipol University, Istanbul 34214, Turkey; dr.yelizcicek@gmail.com (Y.Ç.); mahirkapmaz@yahoo.com (M.K.); 2 Epidemiology PhD Program, Graduate School of Health Sciences, Istanbul Medipol University, Istanbul 34810, Turkey; okanderin@gmail.com; 3 Department of Infectious Diseases and Clinical Microbiology, Istanbul Şişli Hamidiye Etfal Training and Research Hospital, Istanbul 34396, Turkey; 4 Department of Infectious Diseases and Clinical Microbiology, Faculty of Medicine, Bursa Uludağ University, Bursa 16059, Turkey; halis@uludag.edu.tr (H.A.); uguronal@uludag.edu.tr (U.Ö.); egemenozdemir@uludag.edu.tr (E.Ö.); 5 Department of Infectious Diseases and Clinical Microbiology, Ankara Training and Research Hospital, Ankara 06230, Turkey; cigdemhatip@yahoo.com (Ç.A.H.); tuncergunay@yahoo.com (G.T.E.); 6 Department of Infectious Diseases and Clinical Microbiology, Izmir Katip Celebi University, Ataturk Training and Research Hospital, Izmir 35360, Turkey; dr.alpersener@gmail.com (A.Ş.); dr.leylaakgul@gmail.com (L.A.); 7 Department of Infectious Diseases and Clinical Microbiology, Faculty of Medicine, Balikesir University, Balikesir 10145, Turkey; yesim.alpay@hotmail.com (Y.Ç.); deryatunaa@hotmail.com (D.T.E.); 8 Department of Infectious Diseases and Clinical Microbiology, Faculty of Medicine, Dicle University, Diyarbakir 21280, Turkey; mkcelen@hotmail.com (M.K.Ç.); nurbahar.oguz@hotmail.com (N.B.O.); 9 Department of Infectious Diseases and Clinical Microbiology, Antalya Vakif Yasam Hospital, Antalya 07060, Turkey; drfigensarigul@yahoo.com; 10 Department of Infectious Diseases and Clinical Microbiology, Bakirkoy Dr. Sadi Konuk Training and Research Hospital, Istanbul 34147, Turkey; drdenizborcak@gmail.com (D.B.); drsevtap@yahoo.com (S.Ş.); 11 Department of Infectious Diseases and Clinical Microbiology, Sancaktepe Sehit Prof. Dr. Ilhan Varank Training and Research Hospital, Istanbul 34785, Turkey; dreyuparslan@hotmail.com; 12 Department of Infectious Diseases and Clinical Microbiology, Faculty of Medicine, Giresun University, Giresun 28200, Turkey; docsinancetin@gmail.com; 13 Department of Infectious Diseases and Clinical Microbiology, Hakkari State Hospital, Hakkari 30000, Turkey; mrym.csr@gmail.com; 14 Department of Internal Medicine, Faculty of Medicine, Istanbul Medipol University, Istanbul 34214, Turkey; alimert@medipol.edu.tr

**Keywords:** long-acting injectable therapy, cabotegravir/rilpivirine, motivational factors, PLWH perspectives, Turkey

## Abstract

*Background and Objectives*: Long-acting cabotegravir and rilpivirine (LA-CAB/RPV) offers an alternative to daily oral antiretroviral therapy (ART) for people living with HIV (PLWH). Although LA-CAB/RPV has been approved in Turkey, the country remains in the pre-rollout period, and national data on patient perspectives are lacking. This is the first nationwide study from Turkey, a setting of increasing HIV incidence, assessing PLWH perspectives on switching to LA-CAB/RPV and the influence of motivational factors on treatment preferences. *Materials and Methods*: A prospective, multicenter, cross-sectional study was conducted across 11 HIV treatment centers representing all regions of Turkey. Virologically suppressed PLWH meeting current eligibility criteria for LA-CAB/RPV were included. Treatment preferences (switch to LA-CAB/RPV or remain on oral ART) and five anticipated motivational domains, namely perceived efficacy, safety, convenience, privacy, and cost, were systematically assessed through structured, face-to-face interviews. *Results*: Among 200 eligible participants, 86% (*n* = 172) preferred switching to LA-CAB/RPV. In all subgroups, LA-CAB/RPV was preferred over oral ART, except for those with no formal literacy. Prior awareness of LA-CAB/RPV was significantly associated with the switching preference (*p* < 0.001), with healthcare providers being the most common source of information, at 45.5% (*n* = 172) (*p* < 0.001). Residential proximity to the healthcare center (*p* = 0.018) and all motivational factors significantly influenced the preference (*p* < 0.05). Notably, when participants who initially chose to remain on oral ART were asked whether they would reconsider switching if injections were administered every six months, overall preference for long-acting therapy increased from 86% to 98%. *Conclusions*: High clinical eligibility and strong acceptability for LA-CAB/RPV were observed among Turkish PLWH. Our findings demonstrate that structured motivational factors significantly influence the treatment preference. Addressing these patient-centered factors and logistical barriers may support the successful integration of long-acting therapies into routine HIV care. Future longer-interval agents may improve patient-centered acceptability.

## 1. Introduction

Human Immunodeficiency Virus (HIV) infection, first identified in the early 1980s, has become a chronic, manageable condition with effective antiretroviral therapy (ART). Despite global improvements in ART access and declining HIV incidence, new diagnoses in Turkey continue to rise [1], underscoring the need for effective, acceptable, and feasible treatment models [2].

Since the discovery of zidovudine (AZT) in 1987 and the introduction of highly active antiretroviral therapy (HAART) in 1996 [3], significant progress has been made in HIV care. The incorporation of Integrase Strand Transfer Inhibitors (INSTIs) since 2007 has further reduced AIDS-related morbidity and mortality by improving sustained viral suppression and life expectancy in people living with HIV (PLWH) [4,5]. Single-tablet regimens have enhanced adherence and quality of life [6,7]; however, lifelong daily dosing can lead to pill fatigue, privacy concerns, stigma, gastrointestinal side effects, and drug–drug interactions [8,9,10,11,12]. These limitations have driven the development of long-acting ART options [12]

Cabotegravir and rilpivirine (LA-CAB+RPV) is a long-acting intramuscular regimen developed with nanomolecular technology. Phase 3 trials have shown that two monthly injections provide similar virologic efficacy and tolerability as oral ART in virologically suppressed adults [13,14,15,16]. Following these results, the European Medicines Agency (EMA) and the US Food and Drug Administration (FDA) approved LA-CAB+RPV in 2020 and 2021, respectively, for eligible PLWH [17,18]. However, the use of this regimen is limited in individuals who are pregnant, co-infected with hepatitis B virus (HBV), infection with certain HIV-1 subtypes, or have suboptimal adherence, due to insufficient data and an increased risk of treatment failure [19,20].

Although LA-CAB+RPV has been authorized for use in Turkey [21], it has not yet been incorporated into routine clinical practice due to the lack of reimbursement coverage by the national health insurance system. This study is the first in Turkey to assess the perspectives of PLWH on this new treatment option during the pre-rollout period.

This study aimed to evaluate the preferences of PLWH regarding switching to LA-CAB+RPV or remaining on their current oral ART and to statistically compare the underlying motivations for these preferences based on a literature-informed framework [22,23,24,25,26].

## 2. Materials and Methods

### 2.1. Study Design and Participants

Our study was a multicenter, cross-sectional, and prospective observational investigation that involved PLWH across 11 healthcare centers located in the seven geographical regions of Turkey. The investigators were physicians working at the participating study centers, all of whom had experience in the clinical management of HIV. The study included volunteer participants who met the inclusion criteria and provided written informed consent after reading the consent form (Supplementary File S1). Participant data were collected by the investigators through patient records and structured face-to-face interviews and were recorded using a standardized data collection form.

#### 2.1.1. Inclusion Criteria

- Age, 18 years or older;
- Receiving a stable oral ART regimen for at least 6 months;
- Having at least two documented HIV RNA measurements <50 copies/mL within the past 6 months.

#### 2.1.2. Exclusion Criteria

According to current HIV treatment and monitoring guidelines [4,19,20], participants were excluded from the study if they had any of the following conditions that pose a risk for treatment failure with LA-CAB/RPV switching:

- A documented or suspected genotypic resistance to non-nucleoside reverse transcriptase inhibitors (NNRTIs) (except K103N) or to INSTIs;
- History of virologic failure with any ART regimen;
- Pregnancy;
- Presence of chronic HBV co-infection;
- Presence of a hip implant or filler;
- Body mass index (BMI) ≥ 30;
- Use of concomitant medications with potential interactions with LA-CAB/RPV;
- Infection with HIV-1 subtype A1 or A6.

### 2.2. Data Collection

Data were collected through structured face-to-face interviews and patient medical records, and were organized into five main sections:

(1) Sociodemographic Data: Age, gender, educational status (participants with no formal literacy were analyzed separately due to distinct patterns in understanding and interpreting informed consent and treatment-related information), place of residence, residential proximity to the healthcare center, housing status, and HIV status disclosure.
(2) Clinical Data: Smoking status and alcohol use, presence or history of substance use disorder, BMI, presence of comorbidities, polypharmacy and the number of drug classes used, route of HIV transmission, duration of ART use, history and number of ART switches, current ART regimen, CD4+ T-cell count at diagnosis and current level, HIV RNA level at diagnosis, history of opportunistic infections, and HIV-associated malignancies.
(3) Awareness of LA-CAB/RPV: Participants were asked whether they had prior knowledge of LA-CAB/RPV therapy and what their sources of information were. Regardless of their initial level of knowledge, all participants were provided with standardized information on LA-CAB/RPV, including evidence-based data and administration procedures, using a uniform treatment information sheet (Supplementary File S2) across all study centers.
(4) Treatment Choice: Participant preference to either continue current oral ART or switch to LA-CAB/RPV.
(5) Treatment Choice Motivations: The role of motivational factors, including perceived efficacy, perceived safety and tolerability, convenience and adherence, privacy and confidentiality, and cost-related concerns, was evaluated in relation to treatment preferences using structured questions designed in line with the literature and formulated to be easily understandable by all participants.

Although psychosocial and economic barriers were not evaluated through separate instruments, these aspects were indirectly addressed within the structured motivational domains and were further explored during interviews when relevant.

In addition, participants who initially preferred to continue daily oral ART were asked whether they would consider switching to a long-acting regimen if a formulation allowing administration every six months became available.

The validity of the structured questions assessing treatment choice motivations (Supplementary File S3) was established through expert review by three specialists, and a pilot test was conducted with ten PLWH. Test–retest reliability was not assessed due to the single-time, decision-oriented design of the study.

### 2.3. Definitions

Documented NNRTI/INSTI resistance was defined according to the Stanford University HIV Drug Resistance Database (HIVDB) algorithm, based on the identification of drug resistance mutations using genotypic methods. Resistance was considered present if at least one mutation associated with low-, intermediate-, or high-level resistance to the relevant drug class was detected [27,28]. In cases where individuals experienced virologic failure while on regular NNRTI or INSTI therapy but genotypic testing was not available, resistance was assumed [4,19,20].

Housing status was categorized as living with family, alone, with a partner, with others, or in a nursing home. HIV status disclosure was defined by the recipients as disclosure to family members, partner(s), close friends, or others.

Residential proximity to the healthcare center was defined according to Türkiye’s administrative boundaries. Participants were classified as residing either in the same district as the healthcare center, in a different district within the same province, in a different province within the same geographical region, or in a different geographical region. Geographical regions were determined based on Türkiye’s official seven-region classification, including Marmara, Aegean, Mediterranean, Central Anatolia, Black Sea, Eastern Anatolia, and Southeastern Anatolia.

Smoking status was assessed based on to the Centers for Disease Control and Prevention (CDC) criteria. Individuals who had smoked fewer than 100 cigarettes in their lifetime were classified as “never smokers,” those who had smoked more than 100 cigarettes but were not smoking at the time of the study as “former smokers,” and those who were actively smoking at the time of the study as “current smokers” [29].

Alcohol use was categorized based on the National Institute on Alcohol Abuse and Alcoholism (NIAAA) criteria as either “low-risk” or “high-risk.” Low-risk drinking was defined as ≤14 drinks per week or ≤4 drinks per day for men, and ≤7 drinks per week or ≤3 drinks per day for women; consumption exceeding these thresholds was considered high-risk alcohol use [30].

Polypharmacy was defined as the concurrent use of five or more medications from different pharmacological classes [31]. BMI was calculated using the CDC Adult BMI Calculator [32].

### 2.4. Statistical Analysis

All analyses were conducted in R (version 4.4.2) [33]. Continuous variables were first assessed for normality using the Shapiro–Wilk test; none met the assumption of normality (all *p* < 0.05). Accordingly, continuous data were presented as the median interquartile range (IQR) and compared between the switch to LA-CAB/RPV and remain on oral ART groups using the two-tailed Mann–Whitney U test. Categorical variables are reported as the number (percentage) and compared between groups using Pearson’s χ^2^ test, with Monte Carlo simulation (10,000 replications) when any expected cell count was <5; Fisher’s exact test was used for 2 × 2 tables with small sample sizes. A two-sided *p*-value < 0.05 was considered statistically significant.

## 3. Results

### 3.1. Study Population and Treatment Preference

A total of 230 PLWH volunteered for the study and met the inclusion criteria. Of these, 13% (*n* = 30) were excluded for meeting at least one exclusion criterion. Specifically, 5.7% (*n* = 13) had chronic HBV co-infection, 3.9% (*n* = 9) had NNRTI resistance (major genotypic mutations: Y188L, Y181C, V179E, E138A, A6V), and 0.4% (*n* = 1) had both NNRTI and INSTI resistance (major genotypic mutations—NNRTI: V179E, Y181C; INSTI: T66A, G118R). Additionally, 3.0% (*n* = 7) were excluded due to a BMI ≥ 30, and 0.9% (*n* = 2) due to treatment failure, poor adherence, and resistance mutations.

The final study population consisted of 200 PLWH who met the eligibility criteria for switching to LA-CAB/RPV. Among them, 86% (*n* = 172) preferred to switch to LA-CAB/RPV, while 14% (*n* = 28) chose to continue their current oral ART regimen.

### 3.2. Sociodemographic, Clinical Characteristics, and Residential Factors

Participants were recruited from seven geographical regions, with the following distribution: Marmara (34.5%, *n* = 69), Central Anatolia (19.5%, *n* = 39), Aegean (12.5%, *n* = 25), Southeastern Anatolia (12.5%, *n* = 25), Mediterranean (10.0%, *n* = 20), Black Sea (6.0%, *n* = 12), and Eastern Anatolia (5.0%, *n* = 10).

**Figure 1:**
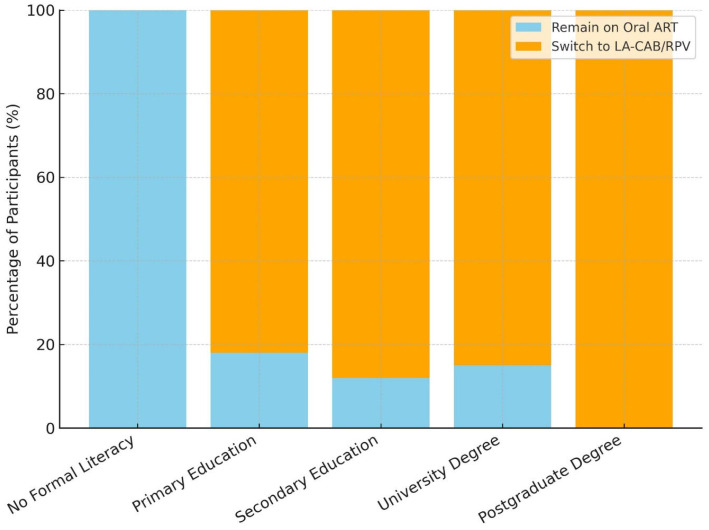
Distribution of treatment preferences by education level (%).

Treatment preferences were compared among sociodemographic and clinical subgroups. In all subgroups except those with no formal literacy, more participants preferred the LA-CAB/RPV switch than remaining on oral ART (Table 1). Although the relationship between educational level and treatment preference was not statistically significant, participants with higher education levels showed a greater tendency to prefer LA-CAB/RPV, as illustrated in Figure 1.

**Figure 2:**
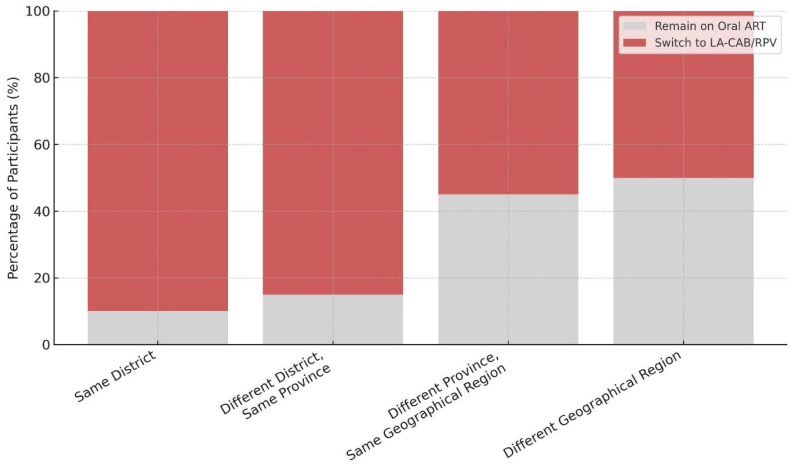
Residential proximity to healthcare center and treatment preference.

Residential proximity to the healthcare center was significantly associated with treatment preference (*p* = 0.018). Preference for LA-CAB/RPV decreased as geographic distance increased (Figure 2).

### 3.3. HIV Transmission, ART Use, and Clinical Follow-Up

Data on HIV transmission routes, ART duration, history of ART switches, and clinical follow-up parameters are summarized in Table 2.

### 3.4. Awareness and Motivational Factors Influencing Treatment Preference

Participants’ awareness of LA-CAB/RPV, information sources, and motivational factors that influenced treatment preferences are presented in Table 3. Awareness of LA-CAB/RPV was significantly associated with a preference for switching (*p* < 0.001), with healthcare providers being the most common source of information (45.5%).

**Figure 3:**
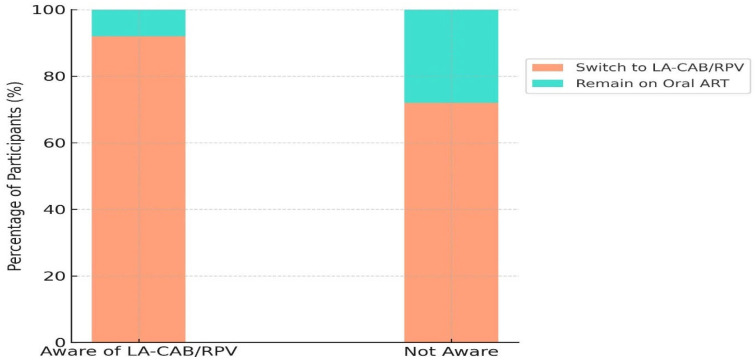
Effect of LA-CAB/RPV awareness on treatment preference.

All motivational factors, including perceived efficacy, safety, convenience, privacy, and cost, were significantly associated with the decision to switch to LA-CAB/RPV (*p* < 0.05), as detailed in Table 3 and Figure 3.

### 3.5. Impact of Hypothetical Six-Monthly Dosing

Among participants who initially preferred to remain on oral ART (*n* = 28), 86% stated they would reconsider switching to LA-CAB/RPV if a six-monthly dosing option became available. Overall, this would raise the preference for LA-CAB/RPV from 86% to 98%.

## 4. Discussion

This study, involving PLWH from all geographical regions of Turkey, demonstrated that the majority were eligible for switching to LA-CAB/RPV and that a considerable proportion expressed a positive attitude towards this therapeutic option. These findings suggest that long-acting injectable therapies are highly feasible within the Turkish HIV population. Similarly, studies conducted in Spain, Italy, United States of America, and Canada have reported high acceptability of LA-CAB/RPV, supporting the consistency of our results with the international literature [34,35,36].

Although not statistically significant, our findings showed a trend toward higher switching rates to LA-CAB/RPV with an increasing education level. When individuals with no formal literacy were excluded, this pattern remained consistent across all sociodemographic subgroups, in line with previous European and Italian studies [35,37].

In this study, participants with an awareness of LA-CAB/RPV had significantly higher switching rates compared to those without awareness. Healthcare providers were the most frequently reported source of information. Significant differences in treatment preference were observed across all information sources. These findings emphasize the key role of healthcare professionals in increasing awareness and promoting the acceptance of long-acting therapy. Similar results have been reported in previous studies. A multicenter European study showed higher switching rates in centers providing educational materials and counselling support [35]. Likewise, a study from Spain demonstrated that individuals actively involved in the information process were more likely to switch to long-acting injectable therapy [38].

Participants who resided farther from treatment centers were significantly less likely to prefer switching to LA-CAB/RPV. Logistical challenges such as transportation difficulties, geographic distance, and limited infrastructure negatively affected treatment decisions. Similar barriers have been highlighted in studies from Italy, Spain, and the United States [34,35,39]. Increasing the uptake of long-acting therapies may be achieved by expanding patient-centered models such as the ‘hub-and-spoke’ system and reducing access-related obstacles [40].

Our study demonstrated that five structured motivational domains were significantly associated with the decision to switch to LA-CAB/RPV. These findings align with prior studies highlighting the critical role of individual preferences in the uptake of long-acting antiretroviral therapies [9,38].

Participants commonly anticipated that LA-CAB/RPV would offer comparable efficacy and improved safety compared to daily oral regimens. This perception is supported by real-world data demonstrating sustained virologic suppression, increased satisfaction, and mild, transient injection site reactions that rarely lead to discontinuation [9,41].

Convenience, adherence, privacy, and reduced disruption of daily life were also frequently cited as key motivations. These domains have previously been shown to improve adherence and quality of life and to reduce treatment fatigue [9,34,38,39,41,42]. Moreover, economic analyses have shown that LA-CAB/RPV is a cost-effective option across different healthcare systems [43,44]; participants in our study also perceived this treatment as more affordable and sustainable.

Our results show that the majority of participants who initially preferred to remain on oral ART would consider switching to a long-acting regimen with six-monthly dosing. Under this hypothetical scenario, overall preference for LA-CAB/RPV would rise to 98%, indicating strong clinical potential for extended dosing regimens. These findings highlight that dosing frequency remains an important determinant of patient preference. Previous research has shown that extending the dosing interval beyond the current monthly or bi-monthly regimens could further improve the acceptability and uptake of long-acting therapies [42]. We believe that developing new long-acting agents with less frequent dosing is a promising approach to enhance treatment acceptability among PLWH.

Our study has several strengths. It represents the first nationwide study from Turkey, a setting of increasing HIV incidence, to investigate patient perspectives on LA-CAB/RPV. Its prospective, cross-sectional, and multicenter design, covering all geographic regions of the country, is a major strength. This broad sampling enhances the representativeness of the findings by reflecting the demographic and clinical characteristics of PLWH across Turkey. Among the excluded individuals, the rates of HBV co-infection and drug resistance were aligned with national epidemiological data [45,46,47,48], which further strengthens the generalizability of our results. Additionally, clearly defined inclusion and exclusion criteria, along with structured, face-to-face data-collection procedures, reinforced both the internal validity and reliability of the study.

Despite its strengths, our study has certain limitations. Due to its cross-sectional design, temporal trends and long-term outcomes could not be assessed. Although LA-CAB/RPV was approved in Turkey, the study was conducted during the pre-rollout period, which may have led participants to base their treatment choices on theoretical expectations rather than practical experience. Additionally, the face-to-face interview method may have introduced a degree of social desirability bias, which could have modestly influenced participants’ reported willingness to switch. Although some socioeconomic indicators, such as employment status, were available, a more comprehensive analysis of income levels, health insurance access, or social support could not be performed due to data limitations. Future prospective studies using real-world data after treatment initiation will be essential to validate our findings. Research exploring healthcare providers’ knowledge and attitudes toward long-acting regimens could also inform system-level implementation strategies.

## 5. Conclusions

In Turkey, PLWH demonstrated high clinical eligibility and strong acceptance of switching to LA-CAB/RPV therapy. Patient-centered factors such as educational level, awareness of treatment, convenience, and privacy emerged as key determinants of treatment preference. However, logistical barriers, particularly geographical distance from healthcare centers, may limit access to this treatment. Our study provides robust nationwide evidence supporting the feasibility of long-acting injectable therapies in HIV care. Strategic priorities should include strengthening patient education, healthcare facilities, and accessibility of services. Integrating LA-CAB/RPV into national HIV treatment programs as a sustainable, patient-centered option could significantly help achieve treatment targets in a country experiencing a rise in new cases.

## Figures and Tables

**Table 1:** Sociodemographic and clinical characteristics of the participants.

	*n* (%) ^1^	Switch to LA-CAB/RPV *n* (%) ^1^	Remain on Oral ART *n* (%) ^1^	*p* Value ^2^
**Age (Median (IQR))**	38.50 (29.00–47.00)	37.50 (29.75–46.25)	41.00 (30.00–52.00	0.367
**Gender (Male)**	177 (88.5)	151 (85.3)	26 (14.7)	0.645
**BMI (Median (IQR))**	25.00 (23.00–27.05)	25.00 (23.00–27.05)	25.30 (22.75–27.03)	0.856
**Educational status**				0.510
No formal education	2 (1)	0 (0)	2 (100)	
Primary education	45 (22.5)	37 (82.2)	8 (17.8)	
Secondary education (high school)	65 (32.5)	59 (90.8)	6 (9.2)	
University degree	84 (42)	70 (83.3)	14 (16.7)	
Postgraduate degree (Master/PhD)	4(2)	4 (100)	0 (0)	
**Place of residence**				0.642
Urban	185(92.5)	158 (85.4)	27 (14.6)	
Rural	15 (7.5)	14 (93.3)	1 (6.7)	
**Residential proximity to the healthcare centre**				0.018
Same district	105 (52.5)	95 (90.5)	10 (9.5)	
Different district, same province	83 (41.5)	70 (84.3)	13 (15.7)	
Different province, same geographical region	6 (3)	4 (66.7)	2 (33.3)	
Different geographical region	6 (3)	3 (50)	3 (50)	
**Housing Status**				0.306
Living with family	132 (66)	113 (85.6)	19 (14.4)	
Living alone	47 (23.5)	41 (87.2)	6 (12.8)	
Living with partner(s)	11 (5.5)	11 (100)	0 (0)	
Living with others (non-family members)	9 (4.5)	6 (66.7)	3 (33.3)	
Residing in a nursing home	1 (0.5)	1 (100)	0 (0)	
**HIV Status Disclosure**				0.643
Family Member(s)	96 (48)	83 (86.5)	13 (13.5)	
Partner(s)	64 (32)	56 (87.5)	8 (12.5)	
Close Friend(s)	36 (18)	29 (80.6)	7 (19.4)	
Other(s)	4(2)	4 (100)	0 (0)	
**Smoking Habit**				0.461
Never Used	89 (44.5)	74 (83.1)	15 (16.9)	
Former Smoker	16 (8)	15 (93.8)	1 (6.2)	
Current Smoker	95 (47.5)	83 (87.4)	12 (12.6)	
**Alcohol Habit**				0.493
Never Used	112 (56)	95 (84.8)	17 (15.2)	
Low-risk Drinking	85 (42.5)	75 (88.2)	10 (11.8)	
Risky Drinking	3 (1.5)	2 (66.7)	1 (33.3)	
**Substance Use Disorder**	18 (9)	17 (94.4)	1 (5.6)	0.467
**Comorbidity**	34 (17)	28 (82.4)	6 (17.6)	0.688
**Polypharmacy**	5 (2.5)	4 (80)	1 (20)	
**Number of Non-ART Drug Classes** **(Median (IQR))**	1.00 (1.00–2.00)	1.00 (1.00–2.00)	2.00 (1.00–3.00)	0.567

^1^: *n* (%) and median (IQR 25–75) results. ^2^: Pearson’s Chi-squared test; Wilcoxon rank sum test; Fisher’s exact test., ART: antiretroviral therapy, LA-CAB/RPV: long-acting cabotegravir rilpivirin injection therapy.

**Table 2:** HIV transmission route, ART use, and clinical follow-up.

	*n* (%) ^1^	Switch to LA-CAB/RPV *n* (%) ^1^	Remain on Oral ART *n* (%) ^1^	*p* Value ^2^
**Transmission Route**				0.371
Sexual (Heterosexual)	94 (47)	78 (83)	16 (17)	
Sexual (Bi/Homosexual)	52 (26)	45 (86.5)	7 (13.5)	
Unknown	46 (23)	43 (93.5)	3 (6.5)	
Percutaneus	7 (3.5)	5 (71.4)	2 (28.6)	
Other (Cupping Therapy)	1 (0.5)	1 (100)	0 (0)	
**Duration of ART Use** **(years (Median (IQR))**	3.00 (2.00–5.00)	3.00 (1.00–5.00)	4.00 (3.00–4.25)	0.240
**History of ART switches**	62 (31)	54 (87.1)	8 (12.9)	0.936
**Number of ART switches** **(Median (IQR))**	1.00 (1.00–1.00)	1.00 (1.00–1.75)	1.00 (1.00–1.00	0.478
**Current Oral ART Regimen**				0.092
BIC/FTC/TAF	129 (64.5)	112 (86.8)	17 (13.2)	
DTG/3TC	26(13)	24 (92.3)	2 (7.7)	
DTG/ABC/3TC	17(8.5)	15 (88.2)	2 (11.8)	
DTG/FTC/TDF	19(9.5)	16 (84.2)	3 (15.8)	
EVG/c/FTC/TAF	9(4.5)	5 (55.6)	4 (44.4)	
**HIV RNA Level (copies/mL) at Diagnosis** **(Median (IQR))**	42,900 (11,400–157,000)	42,600 (12,670–150,200)	54,250 (490–309,750)	0.867
**CD4+ T-cell count (cells/mm^3^) at Diagnosis (Median (IQR))**	416.50 (287.25–583)	417.50 (291–594.5)	391.5 (284.25–483.5)	0.445
**Current CD4+ T-cell count (cells/mm^3^)** **(Median (IQR))**	729 (560.75–921.25)	731 (565.5–921.25)	680 (550–909.5)	0.840
**History of Oppurtunistic Infection**	12 (6)	10 (83.3)	2 (16.7)	0.677
**HIV-associated Malignancy**	5 (2.5)	5 (100)	0 (0)	0.794

^1^: *n* (%) and median (IQR 25–75) results. ^2^: Pearson’s Chi-squared test; Wilcoxon rank sum test; Fisher’s exact test. ART: antiretroviral therapy, LA-CAB/RPV: long-acting cabotegravir rilpivirin injection therapy, BIC: Bictegravir, FTC: Emtricitabin, TDF: Tenofovir Disoproxil Fumarate, TAF: Tenofovir Alafenamide Fumarate, 3TC: Lamivudine DTG: Dolutegravir, ABC: Abacavir, EVG: Elvitegravir c: Cobisistat, HIV: Human Immunodeficiency Virus.

**Table 3:** Awareness of LA-CAB/RPV, sources of information, and reasons for treatment preference.

	*n* (%) ^1^	Switch to LA-CAB/RPV *n* (%) ^1^	Remain on Oral ART *n* (%) ^1^	*p* Value ^2^
**Awareness of LA-CAB/RPV treatment**	161 (80.5)	148 (92)	13 (8)	<0.0001
**Source of Information about LA-CAB/RPV**				<0.0001
Healthcare providers	91 (45.5)	80 (88)	11 (12)	
Other PLWH	7 (3.5)	6 (85.7)	1 (14.3)	
Internet/Social Media	38 (19)	35 (92.1)	3 (7.9)	
Internet/Health information websites	27 (13.5)	22 (81.5)	5 (18.5)	
NGO/Community-based organization	9 (4.5)	7 (77.8)	2 (22.2)	
**Motivations for Treatment Choice**				
Perceived efficacy	169 (84.5)	155 (91.7)	14 (8.3)	<0.0001
Perceived safety/tolerability	144 (72)	130 (90.3)	14 (9.7)	0.0102
Convenience and adherence	176 (88)	157 (89.2)	19 (10.8)	0.0013
Privacy	167 (83.5)	157 (94)	10 (6)	<0.0001
Cost	120 (60)	112 (93.3)	8 (6.7)	0.0006

^1^: *n* (%) and median (IQR 25–75) results. ^2^: Pearson’s Chi-squared test; Wilcoxon rank sum test; Fisher’s exact test. ART: antiretroviral therapy, LA-CAB/RPV: long-acting cabotegravir rilpivirin injection therapy, PLWH: person living with HIV, HIV: Human Immunodeficiency Virus, NGO: Non-Government Organization.

## Data Availability

The datasets generated and/or analyzed during the current study are not publicly available due to patient confidentiality and institutional restrictions. However, anonymized data, with patient identifiers strictly protected, can be made available to the journal editor and peer reviewers upon reasonable request and for the purposes of editorial evaluation.

## Supplementary Materials

The following supporting information can be downloaded at: https://www.mdpi.com/article/10.3390/medicina61081373/s1, File S1: Informed consent form, File S2: LA-CAB/RPV patient information sheet, File S3: Structured assessment document for treatment motivation and preference.

## Abbreviations

The following abbreviations are used in this manuscript:

3TC	Lamivudine
ABC	Abacavir
ART	Antiretroviral Therapy
BMI	Body Mass Index
BIC	Bictegravir
CDC	Centers for Disease Control and Prevention
DTG	Dolutegravir
EMA	European Medicines Agency
EVG	Elvitegravir
FDA	U.S. Food and Drug Administration
FTC	Emtricitabine
GCP	Good Clinical Practice
HBV	Hepatitis B Virus
HIV	Human Immunodeficiency Virus
HIVDB	Stanford University HIV Drug Resistance Database
IQR	Interquartile Range
INSTI	Integrase Strand Transfer Inhibitor
LA-CAB/RPV	Long-Acting Cabotegravir and Rilpivirine Injection Therapy
NGO	Non-Governmental Organization
NIAAA	National Institute on Alcohol Abuse and Alcoholism
NNRTI	Non-Nucleoside Reverse Transcriptase Inhibitor
PLWH	People Living with HIV
R	R Statistical Software
TAF	Tenofovir Alafenamide Fumarate
TDF	Tenofovir Disoproxil Fumarate
WHO	World Health Organization
