# Supplementary material for: Switching to Long-Acting Cabotegravir and Rilpivirine in Turkey: Perspectives from People Living with HIV in a Setting of Increasing HIV Incidence
Source: Medicina (Kaunas). 2025 Jul 29;61(8):1373. doi: 10.3390/medicina61081373 (PMC12388033; doi:10.3390/medicina61081373)
Supplement: Supplementary file 1 [file medicina-61-01373-s001.zip › Supplementary File S1.pdf]

# Supplement S1: Informed Consent Form

You are invited to participate in the study titled *“Perspectives of People Living with HIV in Turkey on Long-Acting Cabotegravir and Rilpivirine Injection Therapy: A Pre-Rollout Feasibility Study.”*

This study aims to evaluate patient perspectives on long-acting injectable HIV treatments. Participation is entirely voluntary.

No treatment or intervention will be administered as part of this study. You will only be asked to answer structured questions prepared by the research team. Your medical records will be reviewed by your treating physician, and your data will be recorded using a coding system to ensure confidentiality.

You have the right to withdraw from the study at any time, without providing any reason and without losing your rights.

All personal identifying information will remain confidential. Even if the results of this research are published, your identity will not be disclosed. Your data will be recorded using a coding system without your full name.

No payments will be made for participation, and no costs will be incurred by you. Completing the questionnaire will be considered your consent to participate.

For further information, you may contact the researcher, Dr. Rıdvan Dumlu, at +90 554 756 11 09.

I have read and/or listened to all the information provided in the informed consent form of this study. The written and verbal explanation regarding the subject and objectives of the study, as stated above, was given to me by the researcher named below. I voluntarily agree to participate in this study, with the full understanding that I may withdraw from the study at any time, with or without providing a reason. I confirm that I am participating in this research freely, without any pressure or coercion.

**Participant:**
Code: ............................................................ Signature: ..................................................
Date: ..................................................
(*Code is formed by the first two letters of the participant's first and last name and the last two digits of the year of birth.*)

If the participant has a legal representative:

**Legal Representative:**
Full Name: .................................................. Signature: ..................................................
Date: ..................................................
(*Code is formed by the first two letters of the representative's first and last name and the last two digits of the year of birth.*)

## Ek 1- Bilgilendirilmiş Gönüllü Olur Formu

## (Turkish Edition)

Sayın Katılımcı,

Sizi 'Türkiye'de HIV ile Yaşayan Kişilerin Uzun Etkili Kabotegravir ve Rilpivirin Enjeksiyon Tedavisine Bakış Açılarının Araştırılması: Tedavi Öncesi Dönemde Bir Fizibilite Çalışması' isimli çalışmaya katılmaya davet ediyoruz. Katıldığınız bu çalışma bilimsel nitelikte bir araştırma olup konusu uzun etkili enjeksiyon tedavilerine hastaların bakış açısının araştırılmasıdır.

Bu araştırma, Medipol Mega Üniversite Hastanesi Enfeksiyon Hastalıkları ve Klinik Mikrobiyoloji servisinden Uzm. Dr. Rıdvan DUMLU tarafınca yürütülmektedir. Araştırmamız kesitsel bir çalışma olup; size çalışma kapsamında herhangi bir tedavi veya invaziv girişim uygulanmayacaktır. Sadece araştırmacının oluşturduğu anket sorularının tematik görüşme ile cevaplamanız istenmektedir. Takip ve tedavi bilgileriniz HBYS üzerinden takip eden hekiminiz tarafınca çalışma veri formuna işlenecek olup, bu işlemler sırasında veri gizliği korunacaktır.

Araştırmaya katılımınız gönüllülük esasına dayanmaktadır. İstediğiniz zaman, herhangi bir cezaya veya yaptırıma maruz kalmaksızın, hiçbir hakkınızı kaybetmeksizin araştırmaya katılmayı reddedebilir veya araştırmadan çekilebilirsiniz. İlgili mevzuat gereğince kimliğini ortaya çıkaracak kayıtların gizli tutulacak olup, kamuoyuna açıklanamayacak araştırma sonuçlarının yayımlanması halinde dahi gönüllünün kimliğinin gizli kalacaktır. Veri formunda açık isim bilginiz asla kullanılmayacak, kodlama sistemi ile bilgileriniz kaydedilecektir.

Araştırmada yer almanız nedeniyle size hiçbir ödeme yapılmayacak ve sizden de hiçbir ücret talep edilmeyecektir. Anketi yanıtlamanız, araştırmaya katılım için onam verdiğiniz anlamına gelmektedir. Araştırma hakkında daha fazla bilgi almak için araştırmacı Rıdvan Dumlu'ya başvurabilir, araştırmacıya günün 24 saatinde 05547561109 numaralı cep telefonundan erişebilirsiniz.
Araştırmanın bilgilendirilmiş gönüllü olur formundaki tüm açıklamaları okudum ve/veya sözlü olarak dinledim. Yukarıda belirtilen araştırmanın konusu ve amacı ile ilgili yazılı ve sözlü açıklamalar, aşağıda belirtilen araştırmacı tarafından tarafıma yapılmıştır. Araştırmaya hiçbir baskı ve zorlama olmaksızın, tamamen kendi rızamla katılmayı kabul ediyorum. Dilediğim zaman gerekçeli veya gerekçesiz olarak araştırmadan ayrılabileceğimi biliyorum.

**Gönüllünün:**
Kodu: .................................................. İmzası: .................................................. Tarih: ..................................................
(*Kodu, ismin ilk iki harfi, soyismin ilk iki harfi ve doğum yılının son iki rakamından oluşur.*)

Yasal temsilcisi olması durumunda:

**Yasal Temsilcinin:**
Adı Soyadı: .................................................. İmzası: .................................................. Tarih:

......................................................

(*Kodu, ismin ilk iki harfi, soyismin ilk iki harfi ve doğum yılının son iki rakamından oluşur.*)
