# Supplementary material for: Switching to Long-Acting Cabotegravir and Rilpivirine in Turkey: Perspectives from People Living with HIV in a Setting of Increasing HIV Incidence
Source: Medicina (Kaunas). 2025 Jul 29;61(8):1373. doi: 10.3390/medicina61081373 (PMC12388033; doi:10.3390/medicina61081373)
Supplement: Supplementary file 1 [file medicina-61-01373-s001.zip › Supplementary File S2.pdf]

Supplement S2: LA-CAB/RPV Patient Information Sheet

Dear Participant,

This document provides information about long-acting injectable HIV treatment with Cabotegravir + Rilpivirine (LA-CAB/RPV). This new treatment has been developed as an alternative to daily oral antiretroviral therapy (ART). The aim is to help you understand your options and make an informed decision.

1. What is this treatment?
LA-CAB/RPV is a long-acting treatment administered by intramuscular injection, containing two antiretroviral agents, cabotegravir and rilpivirine. These medications help control HIV by preventing its replication.

2. Who is eligible?
You may be eligible if:

- Your HIV viral load has been suppressed (<50 copies/mL) for at least 6 months,
- You have no known resistance to the medications,
- You do not have hepatitis B infection or it is under treatment,
- You are not pregnant,
- You can regularly attend appointments.

Your doctor will assess your eligibility based on these criteria.

3. How is it administered?
The treatment is administered as an intramuscular injection every two months, usually at healthcare facilities. Attendance at scheduled appointments is mandatory.

4. Effectiveness
Large clinical trials (FLAIR, ATLAS, ATLAS-2M) have shown that:

- LA-CAB/RPV is as effective as daily oral therapy,
- It maintains viral suppression,
- Treatment failure rates are very low,
- Patient satisfaction with injectable treatment is high.

5. Safety and Side Effects
Most reported side effects are mild and include:

- Pain, redness, or swelling at the injection site,
- Headache,

- Fatigue,
- Muscle aches or flu-like symptoms (rare).

Severe side effects and allergic reactions are very rare. Inform your healthcare provider about any side effects.

6. Important Considerations

- Timely attendance for injections is essential,
- If you miss a dose, contact your doctor (bridging therapy may be required),
- Inform your doctor if you are planning pregnancy or become pregnant,
- Do not stop the treatment without consulting your doctor.

7. Before Starting the Treatment

- Inform your doctor about your HIV treatment history, other medications, and health conditions,
- Your healthcare team will assess your eligibility.

Note: LA-CAB/RPV does not cure HIV, but helps keep the virus under control and reduces transmission risk. With regular treatment and monitoring, you can live a healthy life.

For any questions, consult your healthcare team.

EK 2: LA-CAB/RPV Hasta Bilgilendirme Dökümanı (Turkısh Edition)

Sayın Katılımcı,

Size bu dökümanda, uzun etkili enjeksiyon şeklinde uygulanan HIV tedavisi olan Kabotegravir + Rilpivirin (LA-CAB/RPV) hakkında bilgi verilmektedir. Bu yeni tedavi seçeneği, mevcut günlük ağızdan alınan antiretroviral tedavilere alternatif olarak geliştirilmiştir. Amaç, size uygun tedavi seçeneklerini anlamanızı ve bilinçli karar vermenizi sağlamaktır.

1. Bu Tedavi Nedir?
LA-CAB/RPV, kabotegravir ve rilpivirin adlı iki antiretroviral ilacın kas içine (intramüsküler) enjeksiyon yoluyla uygulandığı bir tedavi biçimidir. Bu ilaçlar, HIV'in çoğalmasını engelleyerek virüsün kontrol altında tutulmasına yardımcı olur.

2. Kimler İçin Uygundur?
Aşağıdaki şartları sağlayan bireyler için uygundur:

- Mevcut tedaviyle en az 6 aydır HIV baskılanmış (HIV RNA <50 kopya/mL),
- HIV ilacına karşı direnç saptanmamış,
- Hepatit B enfeksiyonu olmayan veya tedavi altında olan,
- Gebe olmayan,
- Tedavi randevularına düzenli katılabilecek durumda olan.

Doktorunuz, bu kriterlere göre uygunluğunuzu değerlendirecektir.

3. Uygulama Şekli ve Sıklığı
Tedavi, her 2 ayda bir kalçadan kas içine enjeksiyon şeklinde uygulanır. Enjeksiyonlar sağlık kuruluşlarında yapılır ve randevu günlerine uyulması zorunludur.

4. Etkinlik
FLAIR, ATLAS ve ATLAS-2M gibi büyük klinik çalışmalarda:

- Günlük ilaçlarla aynı etkinlik düzeyine sahip olduğu,
- HIV'in baskı altında tutulduğu,
- Tedavi başarısızlık oranlarının düşük olduğu,
- Enjeksiyon tedavisi alan bireylerin memnuniyetinin yüksek olduğu gösterilmiştir.

5. Güvenlik ve Yan Etkiler
Genellikle hafif ve geçici olan yan etkiler şunlardır:

- Enjeksiyon bölgesinde ağrı, kızarıklık, şişlik,
- Baş ağrısı,

- Yorgunluk,
- Kas ağrısı veya grip benzeri belirtiler (nadir).

Ciddi yan etkiler ve alerjik reaksiyonlar çok nadirdir. Yan etki durumunda sağlık ekibinize başvurunuz.

6. Dikkat Edilmesi Gerekenler

- Randevulara zamanında gitmek çok önemlidir,
- Doz kaçırıldığında doktorunuzla iletişime geçiniz (köprü tedavisi gerekebilir),
- Gebelik düşünüyorsanız veya hamile kalırsanız sağlık ekibinizi bilgilendiriniz,
- Tedaviyi doktor onayı olmadan bırakmayınız.

7. Tedavi Öncesi Yapılması Gerekenler

- HIV tedavi geçmişinizi ve kullandığınız tüm ilaçları doktorunuza bildiriniz,
- Sağlık ekibiniz, uygunluğunuzu değerlendirecektir.

Not: LA-CAB/RPV tedavisi HIV'i tamamen ortadan kaldırmaz, ancak virüsü baskı altında tutarak bulaşma riskini azaltır. Düzenli tedavi ve takip ile sağlıklı bir yaşam sürmek mümkündür.

Herhangi bir sorunuz olursa sağlık ekibinize danışabilirsiniz.
