# Supplementary material for: Switching to Long-Acting Cabotegravir and Rilpivirine in Turkey: Perspectives from People Living with HIV in a Setting of Increasing HIV Incidence
Source: Medicina (Kaunas). 2025 Jul 29;61(8):1373. doi: 10.3390/medicina61081373 (PMC12388033; doi:10.3390/medicina61081373)
Supplement: Supplementary file 1 [file medicina-61-01373-s001.zip › Supplementary File S3.pdf]

Supplement S3: Structured Assessment Document for Treatment Motivation and Preference

This document was used to systematically assess participants' awareness of LA-CAB/RPV treatment, sources of information, and key motivational factors influencing treatment preferences. It covers both participants willing to switch to LA-CAB/RPV and those preferring to remain on daily oral ART.

1a. Awareness of LA-CAB/RPV Treatment

Have you heard about LA-CAB/RPV?

-Yes

-No

1b. If yes, source of information:

-Healthcare providers

-Other PLWH

-Internet/Social Media

-Health information websites

-NGO(Non-gowernment organizations) / Community-based organizations

-Other:

2. Motivational Factors for Switching to LA-CAB/RPV

Participants opting for LA-CAB/RPV selected applicable reasons:

Efficacy: Belief that the injectable treatment will maintain HIV RNA <50 copies/mL.

Safety: Expectation of fewer side effects compared to oral ART.

Adherence: Convenience of not taking daily pills, reducing the risk of missed doses.

Privacy: Not carrying pills helps maintain confidentiality.

Cost: Perception that long-term costs may be lower.

3. Motivational Factors for Remaining on Oral ART

Participants preferring to continue daily oral ART selected applicable reasons:

Efficacy: Concern that LA-CAB/RPV may not maintain viral suppression.

Safety: Fear of injection site reactions or other side effects.

Adherence: Concerns about attending regular clinic visits for injections.

Privacy: Fear of increased personal information exposure during injection visits.

Cost: Belief that oral ART may be more affordable than injectable treatment.

Ek 3: Tedavi Tercihi ve Motivasyonel Faktörler Sorgulama Dökümanı (Turkish Edition)

Bu döküman, katılımcıların LA-CAB/RPV tedavisi hakkında bilgi düzeyini, bilgi kaynaklarını ve tedavi tercihini etkileyen motivasyonel faktörleri sistematik şekilde değerlendirmek amacıyla hazırlanmıştır. Hem LA-CAB/RPV'ye geçmek isteyen hem de mevcut günlük oral ART'yi sürdürmek isteyen katılımcıları kapsamaktadır.

1a. LA-CAB/RPV Tedavisi Hakkında Bilgi Düzeyi

Daha önce LA-CAB/RPV tedavisi hakkında bilginiz var mı?

-Evet

-Hayır

1b. Varsa, bilgiyi nereden edindiniz?

-Sağlık çalışanları

-Diğer HIV ile yaşayan bireyler

-İnternet/Sosyal Medya

-Sağlık bilgi siteleri

-Sivil toplum kuruluşları / Toplum temelli organizasyonlar

-Diğer:

2. LA-CAB/RPV’ye Geçiş Tercih Eden Katılımcıların Motivasyonları

Geçiş yapmayı tercih eden katılımcılar aşağıdaki nedenlerden uygun olanları belirtmiştir:

Etkinlik: Bu tedavinin HIV RNA <50 kopya/mL düzeyini koruyacağına inanıyorum.

Güvenlik: Günlük ilaçlara göre daha az yan etki oluşturacağını düşünüyorum.

Uyum: Günlük ilaç almamak tedaviye uyumumu kolaylaştırır.

Mahremiyet: İlaç taşımaya gerek kalmaması gizliliğimi artırır.

Maliyet: Uzun vadede enjeksiyon tedavisinin daha ekonomik olabileceğini düşünüyorum.

3. Mevcut Günlük Oral ART’yi Sürdürmeyi Tercih Eden Katılımcıların Motivasyonları

Oral ART’ye devam etmeyi tercih eden katılımcılar aşağıdaki nedenlerden uygun olanları belirtmiştir:

Etkinlik: LA-CAB/RPV tedavisinin viral baskıyı sürdüremeyeceğinden endişeliyim.

Güvenlik: Enjeksiyon bölgesinde ağrı veya diğer yan etkilerden kaygılıyım.

Uyum: Düzenli enjeksiyon randevularına gitmekte zorlanabilirim.

Mahremiyet: Enjeksiyon sırasında kişisel bilgilerimin paylaşılmasından çekiniyorum.

Maliyet: Enjeksiyon tedavisinin tabletlerden daha pahalı olabileceğini düşünüyorum.
